# Journeying Through the Hurdles of Gender-Affirming Care Insurance: A Literature Analysis

**DOI:** 10.7759/cureus.36849

**Published:** 2023-03-29

**Authors:** Heli Patel, Justin M Camacho, Neeku Salehi, Romina Garakani, Leigh Friedman, Chris M Reid

**Affiliations:** 1 Department of Medical Education, Nova Southeastern University Dr. Kiran C. Patel College of Allopathic Medicine, Davie, USA; 2 Department of Medicine, Drexel University College of Medicine, Philadelphia, USA; 3 Department of Plastic Surgery, University of California San Diego, San Diego, USA

**Keywords:** gender-affirming care, gender-affirming surgery, policy advocacy, transgender, insurance

## Abstract

Gender-affirming surgery (GAS) has been proven to be successful in the treatment of gender dysphoria. The benefits of providing insurance coverage for transition-related surgeries far surpass the costs of suffering from persistent gender dysphoria, including many positive health outcomes such as decreased rates of substance use, psychiatric illness, and suicide. Despite being deemed a medical necessity, discrepancies in access to treatment and insurance coverage for GAS persist. The purpose of this review is to understand the impact of limited insurance coverage on the well-being of transgender patients. A comprehensive search was conducted utilizing the Preferred Reporting Items for Systematic Reviews and Meta-Analyses (PRISMA) guidelines in SCOPUS and PubMed databases using the terms “insurance” AND “gender affirming surgery.” Articles in non-English languages were excluded. Data related to variations in insurance coverage for GAS in the United States were collected. Of the 67 articles reviewed, 29 met the inclusion criteria. When compared to the general population, individuals who identify as transgender have higher rates of being uninsured as of 2020, with only 30 states in the United States providing insurance coverage for transgender and gender non-binary people. Of the 30 states, only 18 provide coverage for GAS, with chondrolaryngoplasty having the highest prevalence of coverage. As evidenced in our review, the persistence of complex insurance regulations impedes transgender individuals’ access to equitable care. Overall, this literature review elucidates the variability in insurance coverage as it relates to gender-affirming care. Furthermore, this review highlights the need for additional health policy reforms, in addition to improving physician awareness regarding the hurdles of navigating the insurance world as a transgender patient.

## Introduction and background

Gender dysphoria is used to describe the incongruence induced by having a gender identity that differs from the sex assigned at birth. This dissonance can result in significant anxiety and impairment for transgender patients [1]. Currently, there are many treatment options for gender dysphoria, including hormone treatment, counseling, psychotherapy, mental health services, and gender-affirming surgery (GAS) [2]. Gender-affirming care allows transgender patients to resolve their internal conflict, ultimately achieving concordance between self-identified gender, physical appearance, and function.

For more than 80 years, GAS has been pivotal in the treatment regimen for gender dysphoria due to its profound impact on an individual’s quality of life [2,3]. With an estimated 1.4 million adults and 150,000 teenagers self-identifying as transgender, GAS has gained significant momentum [2]. GAS encompasses both genital and non-genital procedures, such as penile and neovaginal reconstruction, chest wall contouring, and facial feminization surgery (FFS) [2].

While the need and positive outcomes of gender-affirming care have been well detailed in the literature, significant barriers continue to pose a threat to individuals seeking care in this field. Transgender individuals are frequently denied access to GAS due to a lack of insurance or financial resources. Moreover, many transgender individuals report experiencing verbal and physical harassment in the clinical setting [4]. While the ruling of the Affordable Care Act (ACA) has made significant strides in increasing access to gender-affirming care, insurance coverage still remains scarce for those pursuing GAS [2]. Therefore, this study aims to provide a comprehensive overview of insurance coverage for gender-affirming interventions and its significance in delivering equitable care.

## Review

Methods

**Figure 1:**
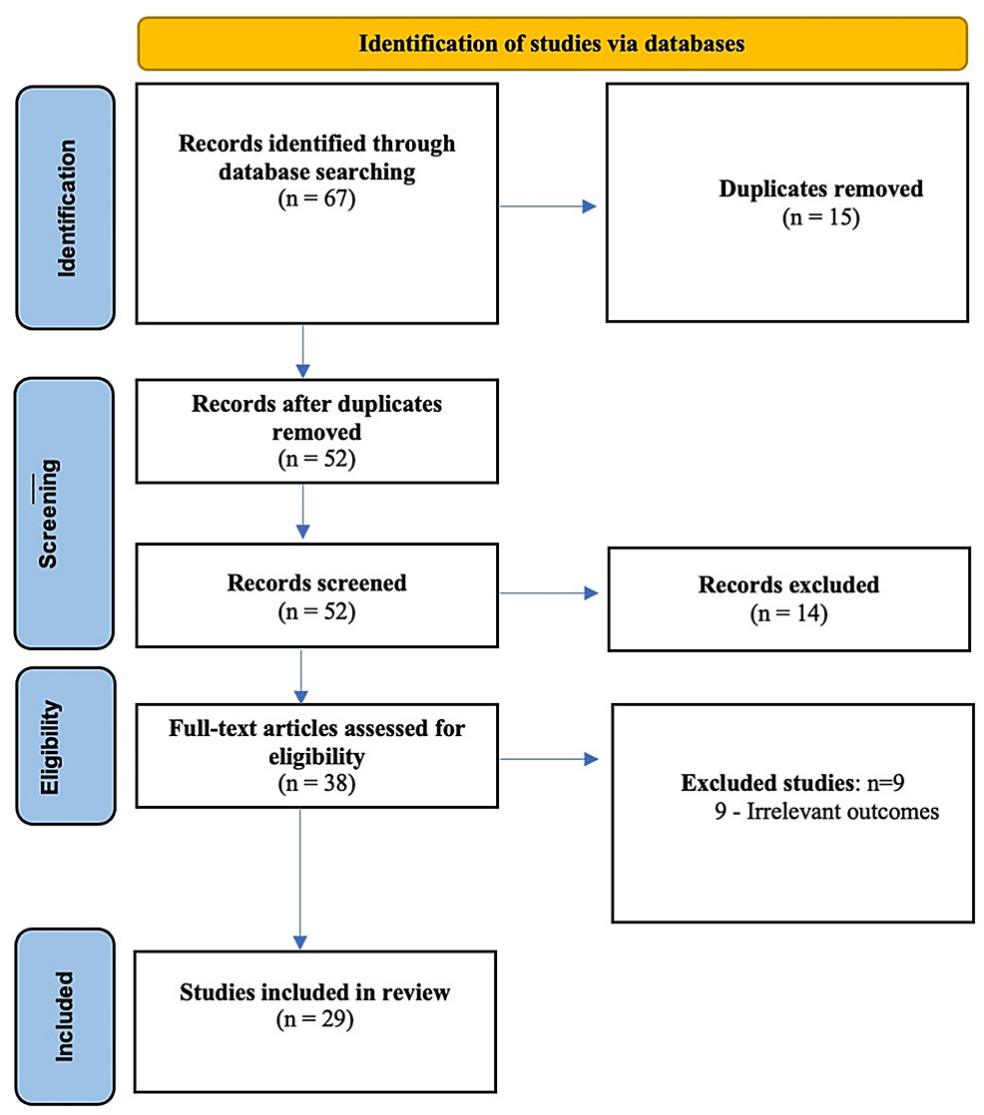
PRISMA protocol for the studies included in this review. PRISMA: Preferred Reporting Items for Systematic Reviews and Meta-Analyses

This review was performed using the PubMed and Scopus databases for articles pertaining to insurance coverage for gender-affirming care. A comprehensive search through the PubMed and Scopus databases was conducted using the terms (insurance) AND (gender affirmation surgery). After screening the titles and abstracts, the articles were identified for free full texts. Additional articles were screened after reviewing references from previously identified articles. The search strategy was designed to include all types of literature, including clinical trials, cohort studies, retrospective studies, case studies, and systematic reviews. Articles published in the English language in any journals were considered. Preferred Reporting Items for Systematic Reviews and Meta-Analyses (PRISMA) guidelines were followed [5]. Data related to the history of insurance for transgender patients and coverage for GAS procedures were extracted (Figure 1). After a review of the free full texts, a total of 67 articles were reviewed in detail. Of these articles, 29 met the inclusion criteria [1-4,6-28]. The remaining articles were excluded due to a lack of information pertaining to insurance coverage for GAS. An ethics statement is not applicable because this study is based exclusively on published literature.

Insurance coverage for gender-affirming surgery

As of 2020, individuals who identify as transgender and gender diverse (TGD) have higher rates of being uninsured, 14%-19% when compared to the general population, 11%-17% [6,7]. While 30 states have detailed explicit coverage for transgender and gender non-binary (TGNB) people as of 2021, only 18 of these states provide coverage for GAS, with 13 states denying such coverage [8]. Therefore, it becomes imperative for plastic surgeons to be cognizant of the insurance coverage for procedures that fall under the umbrella of GAS.

Facial Feminization Surgery

Ngaage et al. [14] elucidate that ancillary procedures, such as FFS, are highly desired by patients as it serves as a treatment for gender dysphoria [9]. However, because FFS is considered a cosmetic surgery rather than a medical necessity, the surgery is not covered by insurance as of 2018 [10]. Despite 19 states excluding coverage for any ancillary procedures, 60% of states provide coverage for chondrolaryngoplasty, which has been found to be the most covered GAS procedure in the United States to date [9].

Hair Restoration and Removal

Hair restoration and removal are also deemed cosmetic surgeries, even though they have been proven to significantly alleviate gender dysphoria for transgender patients [11]. Because the national coverage guidelines for GAS have not been well-established, coverage decisions are made on a local level [9]. As such, most ACA marketplace policies do not mention or provide coverage for hair removal, and thus, many patients end up paying for such procedures out-of-pocket [11]. On the other hand, studies exemplify that individuals who had private insurance companies had a much higher likelihood of receiving coverage for hair removal procedures, with 38.2% of private insurance companies providing some hair removal coverage versus 11.8% of Medicaid policies [9,11].

Chest Reconstruction

Breast and chest reconstructions are also crucial components of an individual’s treatment for gender dysphoria [12]. Nevertheless, insurance coverage for such procedures is insufficient. In 2020, Blasdel et al. analyzed 150 insurance companies in the United States and determined that chest masculinization, chest feminization, and nipple-areola complex reconstruction were covered by 98%, 29%, and 20% of the companies, respectively [12]. The lower coverage rate for chest feminization procedures is because about 75% of health insurers do not deem the procedure to be medically necessary [12]. Insurance companies have also asserted this procedure to be the equivalent of cosmetic breast enlargement procedures in cisgender women and thus continue to deny coverage for transgender patients [2].

Genital Reconstructive Surgery

Over the years, genital surgery has seen an upward trend, with most patients paying out-of-pocket [13]. However, Cohen et al. mention that over 90% of 150 private insurance companies cover procedures with penectomies for vaginal reconstruction, which is the first step in creating female genitalia [2]. These procedures include clitoroplasty, labiaplasty, and vaginoplasty [2]. Cohen et al. also signify that this high coverage rate is due to the consensus that penectomies can help match transgender patients’ genitalia with their gender identity as opposed to the sex assigned to them at birth [2]. On the other hand, less than one-third of insurance companies provided coverage for vulvoplasty, which establishes female genitalia externally but without a vaginal canal [2]. Furthermore, insurance companies continue to classify vulvoplasty as cosmetic and refrain from providing coverage [2]. This discrepancy could be further attributed to the fact that only 17% of insurance companies analyzed by Ngaage et al. established policies aligned with flexible guidelines for genital reconstructive surgery created by the World Professional Association for Transgender Health (WPATH) [14].

Overall, when examining specific gender-affirming procedures covered by states in the United States, chondrolaryngoplasty is the most favorable, followed by facial gender confirmation surgeries, body feminization, hair restoration and removal, and body masculinization [9].

Insurance coverage for gender-affirming interventions

Hormonal Therapy

Gender dysphoria experienced by the transgender population is associated with significant mood disorders that interfere with professional life, personal motivation, healthy relationships, and overall well-being [15]. Starting therapy at an earlier age may mitigate the negative impact on mental health and give rise to improved outcomes; however, due to the financial hardships of obtaining gender-affirming interventions, there is an increased reliance on private insurance [15]. Unfortunately, coverage by private insurance companies is often limited, and health care is restricted to certain providers; thus, the avenue to care depends on the knowledge and competency of these key providers [14]. Upon evaluation of 57 insurance companies by Straumsa et al., only 6% of the companies did not follow WPATH recommendations on the use of hormone therapy before genital reconstructive surgery. However, all companies reached the same consensus regarding the requirement of 12 or more months of hormone therapy before gonadectomy and genital reconstruction [14].

Ensuring access to hormones is an essential harm-reduction strategy because it not only decreases the economic burden for transgender patients but also reduces the risk of non-prescription hormone use [16]. Given the high rates of mental health concerns and significant barriers associated with accessing appropriate hormone therapy, healthcare professionals must work alongside policymakers to reform insurance coverage to be more inclusive of transgender patients [17].

Imaging

As a result of broadening insurance coverage and wider public acceptance of therapies to treat gender dysphoria over the last decade, patients who undergo GAS are more likely to present in non-specialized clinics and emergency departments across the country [2]. Therefore, healthcare professionals must understand the social, mental, physical, medical, and surgical issues unique to this patient population. This also includes radiologists who are not routinely involved in the care of transgender patients [18].

In one study, 10% of TGD patients reported challenges with insurance coverage for imaging services due to their gender identity [19]. To avoid insurance denials for imaging services, such as screening mammograms for transmasculine patients, some TGD patients are forced to mention only the gender assigned at birth on billing forms [19]. Matzanke et al. developed a survey for TGNB patients to explore and evaluate their experiences during imaging encounters [20]. Of the 363 respondents who met the inclusion criteria, 257 (70.8%) reported having had at least one negative imaging encounter. Ultrasound examinations and image-guided procedures contributed to the highest rates of unexpected emotional discomfort (49.1% [109/222] and 38.1% [16/42], respectively) [20]. Additionally, many imaging environments were considered unwelcoming toward TGNB patients, with respondents noting no visible reading materials or other postings that could be considered welcoming to the LGBTQ+ community [21].

Psychological Therapy

The 2015 US Transgender Survey (n = 27,715) conducted by the National Center for Transgender Equality (NCTE) demonstrated that transgender individuals are 12 times more likely to attempt suicide in their lifetime compared to the US general population [22]. Individuals identifying as TGD have also described the lack of health insurance as a notable barrier to utilizing mental healthcare services [23]. Nonetheless, seeing a mental healthcare professional is often required to receive gender-affirming medical treatments such as hormone replacement or GAS.

Ngaage et al. performed a cross-sectional analysis of 57 insurance policies to identify that fewer than two-thirds of the insurers (n = 33) used the WPATH mental health professional qualification criterion [14]. Overall, 33% of insurance companies (n = 17) required at least one referral to be from a mental health professional with a doctoral degree, and 4% (n = 2) required two clinicians with unspecified qualifications [14]. Complex insurance policy requirements pose additional barriers to receiving appropriate gender-affirming medical care and perpetuate high-risk practices such as taking non-prescribed hormones and self-performed surgeries [7].

Fertility Preservation

Gender-affirming procedures and hormone therapy affect the long-term reproductive potential of transgender individuals. Fertility preservation (FP) should ideally be offered before the initiation of gender-affirming hormone therapy (GAHT); however, in the United States, FP is expensive and rarely covered by medical insurance, especially for the transgender community [21,24]. While recent changes in insurance mandates in Connecticut, Delaware, Illinois, Maryland, New Hampshire, New York, and Rhode Island have expanded FP coverage, the implications of these changes for transgender individuals are still unclear [24]. Because medically necessary treatments for gender-affirming interventions compromise fertility, states should consider expanding insurance coverage by recognizing the needs of transgender individuals interested in biological parenthood [24].

Historically, patients who do not identify as cisgender have been denied access to assisted reproductive technology (ART) [25]. In the insurance companies evaluated by Ngaage et al., only 10% of the 52 companies covered FP treatments for transgender patients. An additional 13% considered coverage on a case-by-case basis [14]. As state-level policies on insurance coverage for FP continue to evolve, it is essential for providers of transgender health care to remain aware of medical insurance coverage in their specific state to improve the quality of life of the transgender community [24].

Discussion

Historically, GASs have predominantly been paid out-of-pocket by transgender patients. In 2010, the ACA altered many aspects of the American healthcare system, including the insurance process for transgender patients [6]. With one of its primary goals to expand healthcare insurance for all individuals, the ACA specifically expanded access to medically necessary GAS. Due to the efforts of ACA, a 109% and 392% increase in male-to-female (transfeminine) and female-to-male (transmasculine) procedures was observed three years post-implementation, respectively [6].

In an effort to continue supporting the transgender population, the legislation further banned healthcare discrimination against gender minorities in 2014 [6]. This not only increased insurance coverage for transgender patients but also improved the cultural competence of healthcare professionals surrounding this vulnerable population [10,26]. However, a 2015 survey by Dubov and Fraenkel found that while nearly half of the 27,000 respondents desired some form of GAS, only a quarter had received it. There may be innumerable reasons for this low number, but significant factors that have been underscored are scarcity of qualified physicians, fear of stigma and mistreatment, and lack of insurance coverage [27].

Following the major provisions of the Affordable Care Act in 2016, the Resident Education Center of the American Society of Plastic Surgeons introduced transgender educational modules to improve the quality of care provided to transgender patients within the realms of plastic surgery. Furthermore, in 2019, “Plastic Surgery The Meeting” provided three courses and a facial feminization cadaver laboratory instead of one transgender-related course offered in previous years [6]. As such, there has been a noticeable increase in the educational offerings for plastic surgeons in an effort to enhance their competency in providing GAS. While lack of insurance coverage for GAS has been one of the most significant barriers for transgender patients, a 2021 study highlights that there is a scarcity of plastic surgeons trained and capable of adequately performing GAS [6]. As societal acceptance and prevalence of GAS continue to rise within the United States, trainees in plastic surgery may benefit from exposure to care for transgender patients.

Additionally, in 2016, Section 1557 of the ACA, also referred to as the Health Care Rights Law, was deciphered to ensure that transgender patients receive the same healthcare coverage as cisgender patients [6]. However, several states have actively fought against Section 1557’s protection of transgender people; notably, the Franciscan Alliance in 2016 prohibiting the Health Care Rights Law ruling [6]. In 2020, the enforcement of Title VII of the Civil Rights Act of 1964 further decreased discrimination against transgender people. However, Weignmann et al. speculate that the effects of this ruling are unclear and will likely face pushback from states, synonymous with the outcome of Section 1557 of the ACA [6].

While third-party payers have offered increased coverage for GAS in recent years, over 50% of individuals seeking GAS in 2019 were not granted coverage [6]. To combat this obstacle, the Centers for Medicare and Medicaid Services (CMS) aims to provide coverage for GAS when the procedures are deemed medically necessary [28]. However, decisions on policy coverage are decided on a local level, and, as a result, coverage varies by state for GAS. A 2021 policy review by Gorbea et al. found that of the 50 states in the United States, only 30 addressed transgender non-binary people in their policies [8]. Despite inconsistent national coverage by Medicaid nationally, patients insured by CMS were more likely to have GAS than patients with other insurance plans [18,28].

While previous research clearly highlights transgender patients’ interest in and demand for gender-affirming care, there is a gap in the literature regarding the availability and accessibility of healthcare professions engaged in this field across different geographic regions. Future research is warranted to assess the barriers to accessing gender-affirming care, especially the role of in-state and in-network insurance coverage for specific GAS procedures. Most importantly, our review demonstrates a clear discrepancy between patient interest in gender-affirming care and the insurance coverage for these procedures for historically marginalized transgender patients.

## Conclusions

In tandem with the growing visibility of the transgender community in the United States, there has been an increase in the insurance coverage offered by federal and private insurance companies for transition-related care. Unfortunately, the coverage is often limited, and health care is restricted to certain providers. Thus, multilevel interventions are needed to provide care that affirms the true identity of transgender patients and promotes their well-being, including affordable health insurance coverage, equitable health care, legal protections, and training for healthcare providers. In tandem with the growing visibility of the transgender community in the United States, there has been an increase in the insurance coverage offered by federal and private insurance companies for transition-related care. Unfortunately, the coverage is often limited, and health care is restricted to certain providers. Thus, multilevel interventions are needed to provide care that affirms the true identity of transgender patients and promotes their well-being, including affordable health insurance coverage, equitable health care, legal protections, and training for healthcare providers.

## References

[1] Snelgrove JW, Jasudavisius AM, Rowe BW, Head EM, Bauer GR (2012). "Completely out-at-sea" with "two-gender medicine": a qualitative analysis of physician-side barriers to providing healthcare for transgender patients. BMC Health Serv Res.

[2] Cohen WA, Sangalang AM, Dalena MM, Ayyala HS, Keith JD (2019). Navigating insurance policies in the United States for gender-affirming surgery. Plast Reconstr Surg Glob Open.

[3] El-Hadi H, Stone J, Temple-Oberle C, Harrop AR (2018). Gender-affirming surgery for transgender individuals: perceived satisfaction and barriers to care. Plast Surg (Oakv).

[4] Khan L (2011). Transgender health at the crossroads: legal norms, insurance markets, and the threat of healthcare reform. Yale J Health Policy Law Ethics.

[5] Page MJ, McKenzie JE, Bossuyt PM (2021). The PRISMA 2020 statement: an updated guideline for reporting systematic reviews. BMJ.

[6] Wiegmann AL, Young EI, Baker KE (2021). The Affordable Care Act and its impact on plastic and gender-affirmation surgery. Plast Reconstr Surg.

[7] Murphy AI, Asadourian PA, Marano AA, Rohde CH (2022). Patients and procedures of facial gender confirmation surgery: a NSQIP study. J Craniofac Surg.

[8] Siotos C, Neira PM, Lau BD, Stone JP, Page J, Rosson GD, Coon D (2019). Origins of gender affirmation surgery: the history of the first gender identity clinic in the United States at Johns Hopkins. Ann Plast Surg.

[9] Dubov A, Fraenkel L (2018). Facial feminization surgery: the ethics of gatekeeping in transgender health. Am J Bioeth.

[10] Stowell JT, Grimstad FW, Kirkpatrick DL (2019). Imaging findings in transgender patients after gender-affirming surgery. Radiographics.

[11] Gorbea E, Gidumal S, Kozato A, Pang JH, Safer JD, Rosenberg J (2021). Insurance coverage of facial gender affirmation surgery: a review of Medicaid and commercial insurance. Otolaryngol Head Neck Surg.

[12] Hassan O, Sun D, Jha P (2021). Imaging in gender affirmation surgery. Curr Urol Rep.

[13] Carter SP, Cowan T, Snow A, Cerel J, Tucker R (2020). Health insurance and mental health care utilization among adults who identify as transgender and gender diverse. Psychiatr Serv.

[14] Ngaage LM, McGlone KL, Xue S (2020). Gender surgery beyond chest and genitals: current insurance landscape. Aesthet Surg J.

[15] Thoreson N, Marks DH, Peebles JK, King DS, Dommasch E (2020). Health insurance coverage of permanent hair removal in transgender and gender-minority patients. JAMA Dermatol.

[16] Blasdel G, Nolan IT, Harris AB, Young EI, Hazen A (2020). Limited coverage of gender-affirming breast and chest reconstruction in insurance CPT coding criteria. Plast Reconstr Surg.

[17] Canner JK, Harfouch O, Kodadek LM (2018). Temporal trends in gender-affirming surgery among transgender patients in the United States. JAMA Surg.

[18] Ngaage LM, Knighton BJ, Benzel CA (2020). A review of insurance coverage of gender-affirming genital surgery. Plast Reconstr Surg.

[19] Leinung MC, Urizar MF, Patel N, Sood SC (2013). Endocrine treatment of transsexual persons: extensive personal experience. Endocr Pract.

[20] Stroumsa D, Crissman HP, Dalton VK, Kolenic G, Richardson CR (2020). Insurance coverage and use of hormones among transgender respondents to a national survey. Ann Fam Med.

[21] Grimstad FW, Stowell JT, Gaddis M (2020). Survey of experiences of transgender and gender nonbinary patients during imaging encounters and opportunities for improvement. AJR Am J Roentgenol.

[22] Nahata L, Quinn GP, Caltabellotta NM, Tishelman AC (2017). Mental health concerns and insurance denials among transgender adolescents. LGBT Health.

[23] Stowell JT, Zavaletta VA, Carroll EF, Grimstad FW (2021). Multidisciplinary approach to imaging for gender-affirming surgery: engaging surgeons, radiologists, and patients to ensure a positive imaging experience. Ann Transl Med.

[24] Al-Hiraki S, Nichols S, Tran A, O'Connor K (2021). Addressing the disparities transgender patients face in the US health care system. Georgetown Med Rev.

[25] Bradford J, Reisner SL, Honnold JA, Xavier J (2013). Experiences of transgender-related discrimination and implications for health: results from the Virginia Transgender Health Initiative Study. Am J Public Health.

[26] Sermondade N, Benaloun E, Berthaut I (2021). Reproductive functions and fertility preservation in transgender women: a French case series. Reprod Biomed Online.

[27] Parikh N, Chattha A, Gargollo P, Granberg C (2021). Fertility preservation: a tale of two testicles. Urology.

[28] Kyweluk MA, Reinecke J, Chen D (2019). Fertility preservation legislation in the United States: potential implications for transgender individuals. LGBT Health.

